# Cisplatin Reduces the Frequencies of Radiotherapy-Induced Micronuclei in Peripheral Blood Lymphocytes of Patients with Gynaecological Cancer: Possible Implications for the Risk of Second Malignant Neoplasms

**DOI:** 10.3390/cells10102709

**Published:** 2021-10-09

**Authors:** Aneta Węgierek-Ciuk, Anna Lankoff, Halina Lisowska, Piotr Kędzierawski, Pamela Akuwudike, Lovisa Lundholm, Andrzej Wojcik

**Affiliations:** 1 Department of Medical Biology, Institute of Biology, Jan Kochanowski University, Uniwersytecka 7, 25-406 Kielce, Poland; anna.lankoff@ujk.edu.pl (A.L.); Halina.lisowska@ujk.edu.pl (H.L.); andrzej.wojcik@su.se (A.W.); 2 Centre for Radiobiology and Biological Dosimetry, Institute of Nuclear Chemistry and Technology, Dorodna 16, 03-195 Warsaw, Poland; 3 Department of Radiotherapy, Holy Cross Cancer Center, Artwinskiego 3, 25-734 Kielce, Poland; piotr.ke@op.pl; 4 Centre for Radiation Protection Research, Department of Molecular Biosciences, The Wenner-Gren Institute, Stockholm University, 10691 Stockholm, Sweden; pamela.akuwudike@su.se (P.A.); lovisa.lundholm@su.se (L.L.)

**Keywords:** radiotherapy, chemotherapy, chromosomal damage, apoptosis, second primary cancers, second malignant neoplasms, peripheral blood lymphocytes

## Abstract

Gynaecologic cancers are common among women and treatment includes surgery, radiotherapy or chemotherapy, where the last two methods induce DNA damage in non-targeted cells like peripheral blood lymphocytes (PBL). Damaged normal cells can transform leading to second malignant neoplasms (SMN) but the level of risk and impact of risk modifiers is not well defined. We investigated how radiotherapy alone or in combination with chemotherapy induce DNA damage in PBL of cervix and endometrial cancer patients during therapy. Blood samples were collected from nine endometrial cancer patients (treatment with radiotherapy + chemotherapy—RC) and nine cervical cancer patients (treatment with radiotherapy alone—R) before radiotherapy, 3 weeks after onset of radiotherapy and at the end of radiotherapy. Half of each blood sample was irradiated ex vivo with 2 Gy of gamma radiation in order to check how therapy influenced the sensitivity of PBL to radiation. Analysed endpoints were micronucleus (MN) frequencies, apoptosis frequencies and cell proliferation index. The results were characterised by strong individual variation, especially the MN frequencies and proliferation index. On average, despite higher total dose and larger fields, therapy alone induced the same level of MN in PBL of RC patients as compared to R. This result was accompanied by a higher level of apoptosis and stronger inhibition of cell proliferation in RC patients. The ex vivo dose induced fewer MN, more apoptosis and more strongly inhibited proliferation of PBL of RC as compared to R patients. These results are interpreted as evidence for a sensitizing effect of chemotherapy on radiation cytotoxicity. The possible implications for the risk of second malignant neoplasms are discussed.

## 1. Introduction

Due to improvements in cancer therapy and general increase of life expectancy, the prevalence of cancer survivors is increasing [1]. Since ionising radiation and chemotherapy drugs—two therapeutic agents that most cancer patients are treated with—are themselves carcinogens, the improved cancer patient survival is associated with an increased risk of developing second malignant neoplasms (SMN). Indeed, it was estimated that in the years 2005–2009 SMN constituted 19% of all newly diagnosed cancer cases in the USA, with a raising tendency [2]. 

An intriguing question regarding the risk of radiotherapy-induced SMN is related to the shape of the dose–response curve. In 1965, Gray suggested that a competition process exists between the induction of carcinogenic mutations and cell killing with an increasing domination of the latter with increasing dose. Consequently, the shape of the dose–response curve for radiation-induced cancer is bell-shaped [3]. Indeed, such response is observed in animals following whole body exposure [4] and among the survivors of the Hiroshima and Nagasaki atomic bombings [4]. However, the situation is less clear for highly collimated, partial body irradiation, such as delivered during radiotherapy where very high doses can be applied to normal tissues without leading to patient death. Three parallel models exist that describe the relationship between the radiotherapy dose to normal tissue and the risk of cancer: the linear model—assuming a constant increase of risk with dose, the plateau model—assuming a plateau effect, and the competition model—assuming a decline of risk at high doses [5,6]. Investigations on the location of SMN with respect to the therapeutic dose received by a primary tumour failed to show a clear dose–response relationship [7,8]. However, although most SMN were located in the vicinity of the primary tumour, the dose gradient around the planned treatment volume is so steep that the precise allocation of a dose to the site of a SMN is associated with very large uncertainty [9]. It is likely that a single model will not fit all cancer types, as in fact different shapes of the dose-response relationship have been observed for different SMN [10]. 

Chemotherapy is also associated with a risk of SMN, however, the level of risk is not well characterized [11,12,13], especially when chemotherapy and radiotherapy are combined. The drugs differ in their modes of action and specificity to induce various SMN types [14]. Among the various types of chemotherapies, some studies suggest that anthracyclines have a particularly high carcinogenic potential [14,15,16], while others suggest the opposite [12,17]. A recent systematic review revealed that the carcinogenic potential of cisplatin is, contrary to common belief, not higher than that of other chemotherapy drugs [13]. All studies show that the major determinant of the SMN risk is the applied dose of the drug. 

The interpretation of epidemiological studies on the risk of SMN is complicated by several methodological factors such as the lack of appropriate control groups, more diligent case finding and misclassification of SMN [11]. Studies are therefore desirable where the risk of SMN is inferred from the levels of biomarkers measured in exposed tissues [18]. What biomarkers are representative for SMN? Ionising radiation and chemotherapy drugs are potent inducers of chromosomal aberrations and the latter are known to be associated with cancer induction [19]. Hence, the level of chromosomal damage in irradiated cells can be regarded as a meaningful biomarker of cancer risk [18]. Chromosomal damage can be quantified by analysing chromosomal aberrations [20] or micronuclei (MN) [21].

Using the MN assay in peripheral blood lymphocytes (PBL) as a biomarker of cancer risk, we investigated the potential modulatory effect of chemotherapy on radiotherapy-related risk of SMN. The key question was if chemotherapy will increase or decrease the frequencies of radiotherapy-induced MN. Suitable patient groups were cervical cancer patients receiving a combination of radiotherapy and cisplatin and endometrial cancer patients receiving radiotherapy alone. Both patient cohorts received RT to the lower abdomen in similar fractionation schemes. Blood samples were collected shortly before, in the middle and on the last day of radiotherapy and analysed for MN. In addition to measuring the direct impact of radiotherapy, aliquots of blood were ex vivo irradiated with a dose of 2 Gy to test whether therapy modified the ex vivo sensitivity of PBL. In order to measure the impact of cell death on the levels of MN, frequencies of apoptotic cells were morphologically assessed on MN slides [22]. 

## 2. Materials and Methods

### 2.1. Cervix and Endometrial Cancer Patients

**Figure 1:**
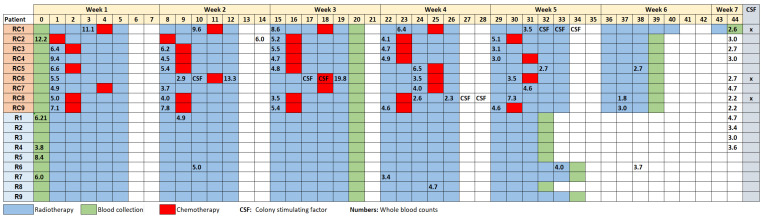
Timeline of treatment and blood collection. Blue and red boxes represent treatments, green boxes represent blood collection. Numbers 0–44 represent days of treatment. Chemotherapy was always given concomitantly to radiotherapy. One row represents one patient. Numbers in the patient rows represent white blood cell counts in 10^9^/L.

All patients were treated in the Holy Cross Cancer Centre, Kielce, Poland. The cohort consisted of nine cervical cancer patients who received radiotherapy plus chemotherapy (RC group) (average age 56.7 ± 10.2) and nine endometrial cancer patients (average age 62.3 ± 9.1) who received radiotherapy alone (R group). All diagnoses were verified by histological examination of the tumours based on the FIGO (International Federation of Gynecology and Obstetrics) classification. Information on age of patients, FIGO staging, dose and number of fractions, number of chemotherapy cycles and colony stimulating factors (CSF) is given in Table 1. A scheme showing the timing of treatment and treatment components is shown in Figure 1. 

Radiotherapy consisted of whole pelvis external beam irradiation. RC patients received 1.8 Gy per fraction, R patients 2 Gy per fraction, 5 days weekly. The total doses and doses per fraction received by each patient are given in Table 1. Radiotherapy was carried out with a Siemens ARTISTE 160 MLC linear accelerator, photon energy of 15 MeV. The technique was 3D conformal radiation therapy with block or multileaf collimators. The patients were exposed to 3–6 beams of various field areas. On average, the field areas of RC patients were somewhat larger than those of R patients (298 ± 65 cm^2^ and 231 ± 46 cm^2^, respectively). 

In addition to radiotherapy, RC patients received cisplatin administered intravenously at a dose of 40 mg/m^2^ given in a 2-h infusion with fitting hydration (cisplatin in 500 mL of normal saline preceded by 500 mL of normal saline and followed by 50 mL of 5% glucose with 2 mg of furosemide). Antiemetic therapy, including ondansetron (8 mg i.v.) or tropisetron (5 mg i.v.) was routinely given before cisplatin infusion. Patients received a minimum of three and a maximum of five cycles of cisplatin. White blood cell (WBC) counts were assayed at several occasions. CSF were given if the WBC fell below 2.0 × 10^9^/L.

All patients gave their informed consent for the analysis. The study was approved by the Ethics Committee from the Cancer Center and Institute of Oncology in Warszawa, Poland (reference number 6/2007).

### 2.2. Blood Sample Processing and Ex Vivo Irradiation

Blood samples were collected from the cancer patients before the onset of radiotherapy, 3 and 5 or 6 weeks after initial radiotherapy treatment (Figure 1). Blood was collected into heparinized (10–20 U/mL) Greiner bio-one tubes (Greiner Bio-One GmbH, Frickenhausen, Germany). Two test tubes were set up from each patient: one as a control sample and one for irradiation. Irradiation of the blood sample took place on the same day as blood collection. The test tube was placed inside a parafilm bolus and exposed at room temperature to 2 Gy of gamma radiation (^60^Co, dose rate 1.13 Gy/min, Siemens Theratron Elite 80, Forchheim, Germany). Blood samples were then processed for analysis of micronuclei and apoptosis. 

### 2.3. Cell Culture and the Micronucleus Assay

Whole blood cultures were set up by adding 0.5 mL of whole blood to 4.5 mL of RPMI 1640 medium (Sigma Aldrich, St. Louis, MO, USA), supplemented with 20% fetal bovine serum (Sigma Aldrich), 10 µg/mL PHA (Gibco, Thermo Fisher Scientific Inc., Waltham, MA, USA), 2 mM L-glutamine, 100 U/mL penicillin and 100 µg/mL streptomycin (Gibco), and cultured at 37 °C and 5% CO_2_ for a total time duration of 72 h. Cytochalasin B (Sigma Aldrich), at a final concentration of 10 μg/mL was added 44 h after incubation started in order to block cytokinesis and obtain binucleated cells. Earlier studies identified this time point of cytochalasin B addition as optimal in that minimal division occurs before [23] and has been used by us and others before [24,25]. After subsequent 28 h the cells were harvested according to the protocol of [21]. In brief, following centrifugation and mild hypotonic treatment (0.14 M KCl) (Sigma Aldrich) for 5 min., cells were washed twice with fixative 1 (0.9% NaCl, methanol and acetic acid (13:12:3) (Sigma Aldrich) and subsequently with fixative 2 (methanol/acetic acid, 4:1). The cells were washed in fixative 2 until the supernatant was clear then dropped onto pre-cleaned microscope slides (Menzel-Glasser, Braunschweig, Germany) and air dried. The slides were stained for 10 min with 5% Giemsa (Merck, Darmstadt, Germany), diluted in phosphate buffer (0.06 M Na_2_HPO_4_ and 0.06 M KH_2_PO_4_, pH 6.8) (Sigma Aldrich), washed with distilled water and air dried. Before scoring slides were coded and scored blind at 400× magnification using a Nikon Eclipse 400 microscope (Tokyo, Japan). For each experimental point 1000 binucleated cells (BNC) were counted and 500 mono and polynucleated cells were scored for the binucleation index. The criteria for the identification of MN were according to [21]. 

### 2.4. Morphological Observation of Apoptotic Cells

Apoptotic cells were counted on the same slides as micronuclei according to criteria published by [26]. Analysis was carried out under a light microscope (Nikon Eclipse 400, Nikon, Tokyo, Japan). For each experimental point 500 cells were counted.

### 2.5. Statistical Analysis

The 95% confidence intervals for frequencies of micronuclei, percent binucleated cells and apoptosis were calculated based on Gaussian statistics. Coefficients of variation were calculated by dividing the standard deviation by the respective mean. Correlations between individual MN frequencies were calculated using the Pearson product–moment correlation method. In addition, correlations between mean MN frequencies, mean percent BNC and mean percent apoptosis were calculated using the Pearson product–moment correlation method. Differences between R and RC patient groups for micronucleus frequencies, binucleation indices and apoptosis frequencies were analysed by One-Way Repeated Measures Analysis of Variance (ANOVA) with the Holms-Sidak test for pairwise multiple comparison testing. All statistical analyses were carried out using SigmaStat 3.5 (Systat Software Inc, San Jose, CA, USA, available online: https://sigmastat.software.informer.com/3.5/, accessed on 9 September 2021). To this end the series of average values per group and treatment type (therapy alone and therapy plus 2 *Gy* ex vivo exposure) were compared with each other. *p* values < 0.05 were considered significant. Optimal fits of mean results per patient group were identified with the help of CurveExpert Professional 2.6 (Hyams Development, Chattanooga, TN, USA, available online: https://www.curveexpert.net, accessed on 9 September 2021). 

## 3. Results

**Figure 2:**
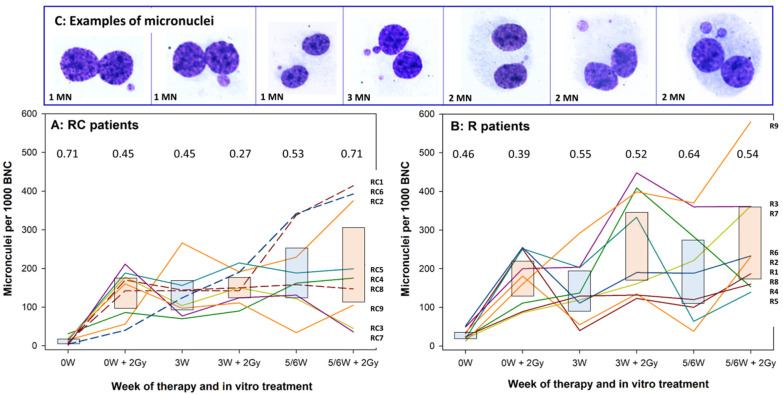
Individual micronucleus frequencies in lymphocytes of RC (**A**) and R (**B**) patients. Each line represents a single donor. Lines are shown to facilitate identification of donors and not to represent a time response. Boxes show 95% confidence intervals of the respective mean value. Blue boxes: values induced by therapy alone. Red boxes: values induced by therapy plus 2 Gy ex vivo irradiation. Dashed lines represent patients RC1, RC6 and RC8 who received colony stimulating factors (see Figure 1). 2 Gy designates samples irradiated under ex vivo conditions. Numbers above each treatment represent coefficients of variation. RC: patients receiving radiotherapy + chemotherapy; R: patients receiving radiotherapy alone; W: week of treatment. Patient numbers are given on the right Y axes in the order of the last treatment (5/6W + 2Gy). (**C**): exemplary images of binucleated cells with micronuclei.

Individual levels of micronucleus frequencies in lymphocytes of RC patients are shown in Figure 2A and of R patients in Figure 2B. Data points of each patient are connected and symbols omitted to facilitate tracing of each individual. Ninety-five percent confidence intervals of the mean are shown as blue boxes for MN frequencies induced by therapy alone and by red boxes for MN frequencies induced by therapy plus 2 Gy ex vivo irradiation. Coefficients of variation for each blood collection time point are shown as numerical values. Strong individual variation in response to therapy and the ex vivo exposure was seen. None of the RC or R patients showed a consistently low or high level of MN throughout the course of whole therapy, indicative of a defined level of intrinsic radiation sensitivity. However, trends could be detected for some patients during second half of therapy: high MN frequencies in patients RC1, RC6, RC2, R3 and R9 and low frequencies in patients RC3, RC7, RC9, R1 and R8. Two out of three RC patients who received CSF showed the highest MN frequencies at 5/6W and 5/6W + 2 Gy (RC1 and RC6) while the MN frequencies of the third (RC8) were at the average level. No consistent differences in the levels of coefficients of variation were detected between RC and R patients.

**Figure 3:**
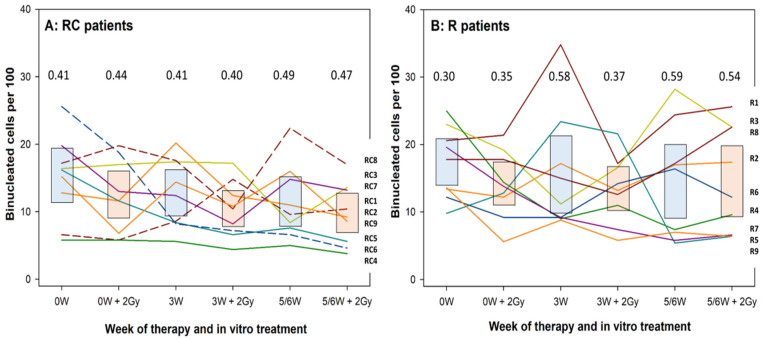
Individual values of cell proliferation marker in lymphocytes of RC (**A**) and R (**B**) patients. Each line represents a single donor. Lines are shown to facilitate identification of donors and not to represent a time response. Boxes show 95% confidence intervals of the respective mean value. Blue boxes: values induced by Therapy alone. Red boxes: values induce by therapy plus 2 Gy ex vivo irradiation. Dashed lines represent patients RC1, RC6 and RC8 who received colony stimulating factors (see Figure 1). 2 Gy designates samples irradiated under ex vivo conditions. Numbers above each treatment represent coefficients of variation. RC: patients receiving radiotherapy + chemotherapy; R: patients receiving radiotherapy alone; W: week of treatment. Patient numbers are given on the right Y axes in the order of the last treatment (5/6W + 2Gy).

The individual levels of cell proliferation (expressed as percent binucleated cells) are shown for RC patients in Figure 3A and of R patients in Figure 3B. Data points of each patient are connected, and symbols omitted to facilitate tracing of each individual. Ninety-five percent confidence intervals of the mean are shown as blue boxes for MN frequencies induced by therapy alone and by red boxes for MN frequencies induced by therapy plus 2 Gy ex vivo irradiation. As for MN, strong individual variation in response to therapy and the ex vivo exposure was seen. Except for donor RC4, RC or R patients did not show a consistently low or high level of cell proliferation throughout the course of therapy, indicative of a defined level of radiation sensitivity. No consistent differences in the levels of coefficients of variability were detected between RC and R patients.

**Figure 4:**
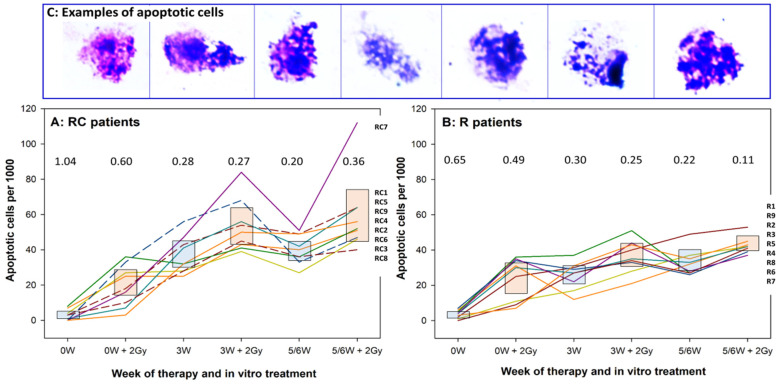
Individual levels of apoptosis in lymphocytes of RC (**A**) and R (**B**) patients. Each line represents a single donor. Lines are shown to facilitate identification of donors and not to represent a time response. Boxes show 95% confidence limits of the respective mean value. Blue boxes: values induced by Therapy alone. Red boxes: values induce by therapy plus 2 Gy ex vivo irradiation. Dashed lines represent patients RC1, RC6 and RC8 who received colony stimulating factors (see Figure 1). 2 Gy designates samples irradiated under ex vivo conditions. Numbers above each treatment represent coefficients of variation. RC: patients receiving radiotherapy + chemotherapy; R: patients receiving radiotherapy alone; W: week of treatment. Patient numbers are given on the right Y axes in the order of the last treatment (5/6W + 2Gy). (**C**): exemplary images of apoptotic cells.

The individual levels of apoptosis are shown for RC patients in Figure 4A and of R patients in Figure 4B. Data points of each patient are connected and symbols omitted to facilitate tracing of each individual. Ninety-five percent confidence intervals of the mean are shown as blue boxes for MN frequencies induced by therapy alone and by red boxes for MN frequencies induced by therapy plus 2 Gy ex vivo irradiation. Coefficients of variation for each blood collection time point are shown as numerical values. The three RC patients who received CFS were not distinctive. Similarly as for MN and proliferation, individual variation in response to therapy and the ex vivo exposure was seen, but the extent of variability was lower than for the other endpoints, as demonstrated by the generally lower levels of coefficients of variation. 

**Figure 5:**
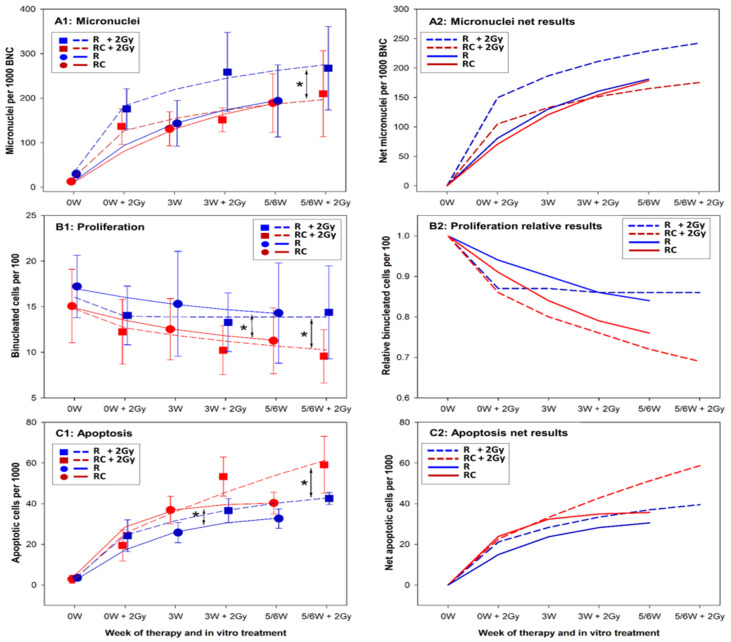
Mean results for RC and R patients. (**A**) panels: micronucleus frequencies, (**B**) panels: proliferation marker, (**C**) panels: apoptosis levels. Right panels (marked 2) show the net or relative fits to facilitate following the kinetics of response. Round symbols and solid lines demonstrate the effect of therapy alone. Square symbols and dashed lines demonstrate the effect of therapy plus 2 Gy ex vivo irradiation. RC: patients receiving radiotherapy + chemotherapy; R: patients receiving radiotherapy alone; W: week of treatment. Asterisks indicate significant differences between the respective curves.

The mean results per patient group are shown in Figure 5(A1) for MN, Figure 5(B1) for proliferation and Figure 5(C1) for apoptosis. To visualise differences between treatments, data were fitted and lines representing the fits were drawn to connect data points describing the effect of therapy alone and therapy plus 2 Gy ex vivo exposure. The spontaneous values (at 0W) of MN, binucleated cells and apoptosis differed slightly between RC and R and in order to follow the impact of treatment, net fits to the data were calculated by subtracting the control values for MN and apoptosis. The relative fits of proliferation results were calculating by expressing the treatment values as fraction of the respective control. The results are shown in Figure 5(A2) for MN, Figure 5(B2) for proliferation and Figure 5(C2) for apoptosis.

The MN frequencies were fitted to the saturation growth rate model:

(1) $$y=\frac{ax}{\left(b+x\right)}$$

where *x* is the time of blood collection and *a*, *b* are fitting coefficients. The fits are shown in Figure 5(A1). MN frequencies increased during therapy. Therapy alone induced the same MN frequencies in lymphocytes of RC and R patients. 2 Gy ex vivo exposure lead to significantly higher MN frequencies of R patients as compared to RC patients. The trend was seen already at 0W + 2Gy and widened with therapy progression. 

The percent values of binucleated cells were fitted to the logistic regression model:

(2) $$y=\frac{a}{\left(1+be^{-cx}\right)}$$

where *x* is the time of blood collection, *a*, *b*, *c* are fitting coefficients and e is Euler’s constant (2.72). The proliferation of lymphocytes decreased during therapy (Figure 5(B1,B2)). Lymphocytes of RC patients proliferated significantly less than those of R patients both when analysed in samples exposed to therapy alone and therapy plus 2 Gy ex vivo exposure and the effect increased with therapy progression indicating a progressive impact of cisplatin. The effect was somewhat stronger in cells exposed ex vivo to 2 Gy.

The frequencies of apoptotic cells were fitted to the exponential association model:

(3) $$y=a\left(1-e^{-bx}\right)$$

where *x* is the time of blood collection, *a*, *b* are fitting coefficients and *e* is Euler’s constant (2.72). Therapy increased the level of apoptosis (Figure 5(C1,C2)) with significantly more apoptotic cells in PBL of RC patients as compared to R. The difference remained stable with therapy progression. Following the ex vivo dose of 2 Gy, more apoptotic cells were observed in PBL of RC patients as compared to R, but only at 3W and 5/6W collection times. The difference between both patient groups widened with time of therapy indicating a progressive impact of cisplatin.

**Figure 6:**
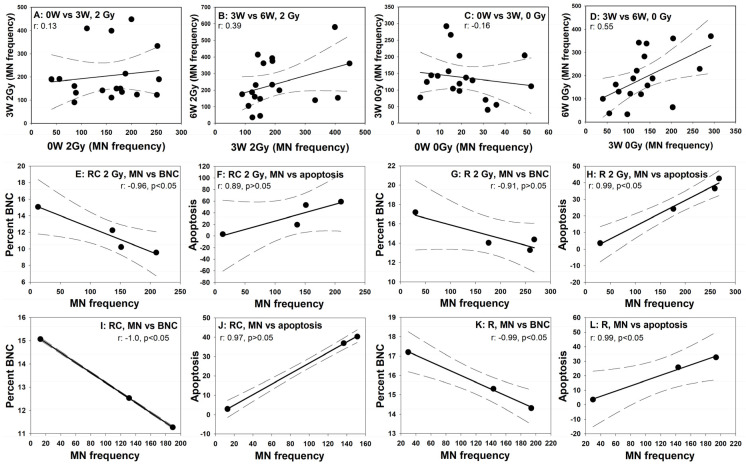
Correlations. Panels (**A**–**D**): correlations between individual micronucleus frequencies observed in all 18 donors at different blood collection time points. Panels A and B: micronuclei induced by therapy + 2 Gy. Panels (**C**) and (**D**): micronuclei induced by therapy alone. Panels (**E**–**L**): correlations between the mean frequencies of MN, percent BNC and percent apoptosis. Panels (**E**–**H**): results after 2 Gy ex vivo irradiation (RC patients: (**E**,**F**), R patients (**G**,**H**)), panels (**I**,**L**): results after therapy alone (RC patients: (**E**,**F**,**I**,**J**), R patients (**K**,**L**)). W: week of treatment. Dashed lines symbolise 95% confidence intervals.

In order to verify how far the level of MN frequency was specific for a particular patient, correlation analyses were carried out between MN frequencies scored at the different RT time points. To this end values from RC and R patients were pooled. The correlations between MN frequencies from the adjacent RT time points 0W:3W and 3W:6W are shown in Figure 6. No significant correlation was detected between any time point combinations although positive trends were noted for MN frequencies in PBL of R and RC patients between 3W and 6W (Figure 6D). The correlation coefficients (r) were generally very low.

Correlations between the mean frequencies of MN, percent BNC and percent apoptosis were calculated and the results are shown in Figure 6, panels E-L. Generally, very good negative correlations were observed between MN frequencies and cell proliferation. In addition, good positive correlations were observed between the MN frequencies and the level of apoptosis. The correlation coefficients (r) were very high (around 0.9 or −0.9) with *p*-values oscillating around the significance level of 0.05. 

## 4. Discussion

The aim of the study was to compare, in PBL of gynaecological cancer patients, the levels of chromosomal damage and apoptosis induced by radiotherapy alone (R patients with endometrium cancer) and radiotherapy plus cisplatin chemotherapy (RC patients with cervical cancer). In addition, the impact of both therapy entities on the ex vivo radiosensitivity of PBL was analysed. The results show that the combination of radiotherapy and chemotherapy did not induce more chromosomal damage than radiotherapy alone, despite the fact that the tumour cure doses received by the RC patients were generally higher than those received by the R patients, the radiation field areas were larger and the patients received cisplatin which is a known inducer of cytogenetic damage (discussed in more detail below). More remarkably, the ex vivo dose of 2 Gy induced fewer MN in PBL of RC patients as compared to R patients. The MN results were inversely correlated with the levels of apoptosis, suggesting that the sparing effect of chemotherapy on the level of chromosomal damage is due to the sensitisation of PBL towards radiation-induced cell death. 

The therapeutic effect of cisplatin is based on its ability to induce DNA adducts with consecutive purine bases in the DNA, leading to either intra-strand or inter-strand crosslinks in the DNA, ultimately blocking DNA replication. Cisplatin-induced DNA damage is primarily repaired via nucleotide excision repair (NER) and homologous recombination [27]. Crosslinks halt DNA replication, and, if unresolved, force cells into apoptosis [27,28]. There is clear evidence that cisplatin induces chromosomal damage in cells exposed both under ex vivo [29] and in vivo conditions [30]. What is interesting from the perspective of the present study is its possible interaction with ionising radiation. Is the interaction positive, potentiating the radiation effect or negative, attenuating the effect? The answer to this question depends on the type of effect.

In human PBL cisplatin was shown to inhibit the processing of radiation-induced DNA double strand breaks leading to reduced formation of radiation-induced repair foci [31]. In cancer cells, the combination of cisplatin and radiation has been shown to have additive and/or supra-additive interactions, predominantly observed as increased cell killing [32]. In human lung carcinoma cells cisplatin did not potentiate the level of radiation-induced chromosomal aberrations but significantly reduced cell survival by inducing apoptosis [33]. A similarly potentiating effect on cell killing by radiation was demonstrated using multicellular spheroids derived from squamous cell carcinoma cell line HN-1 [34]. The interaction was attributed to a radiosensitisation of cancer cells by cisplatin via inhibition of DNA damage repair [35,36]. Other mechanisms such as cisplatin-mediated cell cycle redistribution, the formation of toxic platinum intermediates, as well as the inhibition of the non-homologous end joining (NHEJ) have been described as plausible mechanisms of cisplatin-mediated radiosensitisation towards cell death [37,38]. Proximity of cisplatin-induced DNA adducts have been shown to inhibit the translocation of the Ku DNA binding subunit of DNA-dependent protein kinase (DNA-PK) [39]. Studies in squamous cell carcinomas of the head and neck have shown that concomitant chemoradiation treatment with cisplatin and radiation inhibits sublethal radiation damage repair [40,41] via the inhibition of NHEJ [37,39] leading to cell death. 

The results discussed above demonstrate that cisplatin interacts with radiation, potentiating cell killing. When highly damaged cells are eliminated from a pool of cells, the frequency of chromosomal damage in surviving cells is reduced. In PBL of RC patients studied by us, lower frequencies of MN induced by the ex vivo dose of 2 Gy were observed as compared to R patients. The reduced MN frequencies were accompanied by reduced proliferation and increased apoptosis, strongly suggesting a sensitizing effect of cisplatin on the cytotoxic effect of the ex vivo irradiation. Did cisplatin reduce the level of cytogenetic damage induced by radiotherapy? A problem with answering this question is that RC and R patients differed not only in the cisplatin treatment. On average, total therapy doses were higher and radiation fields larger in RC patients, and both factors are known to potentiate the level of cytogenetc damage in PBL of patients [42]. Thus, a direct comparison of radiotherapy-induced MN frequencies in both patient groups is not possible. However, there is reason to assume that if there was no cisplatin treatment, the frequencies of MN in PBL of RC patients would have been higher than in R patients. The fact that this was not the case can be interpreted as indirect evidence that cisplatin also decreased the frequency of MN induced by radiotherapy. This interpretation of the results, although not directly provable, is substantiated by the observed lower proliferation and higher apoptosis levels in PBL of RC patients, similar as induced by the ex vivo irradiation.

Considering that chromosomal damage can be regarded as a biomarker of cancer risk [19], the results indicate that cisplatin chemotherapy could actually protect patients against radiotherapy-induced SMN, at least of the lymphatic system, by selectively eliminating radiation-damaged cells. How far is this conclusion reflected in results of epidemiological investigations on the risk of SMN? Ideally, the focus should be on the incidence of leukaemias and lymphomas among gynaecological patients treated by cisplatin. The problem is that gynaecological cancers occur relatively late in life (the mean age of all patients included in the present study was 59.5) where the risk of SMN is lower [43]. Moreover, although a number of studies have been carried out on the risk of SMN following radiotherapy of gynaecological cancers [44,45,46,47], the impact of chemotherapy has, as to our knowledge, not been investigated. Similarly, we could not find any data on the impact of cisplatin alone on the risk of SMN induced by radiotherapy. Moreover, lymphomas and leukaemias are not among the common SMN in this patient group. In view of this we screened the literature for evidence of interaction of any form of chemotherapy with radiotherapy on the risk of any SMN. 

A number of investigations do exist on the impact of various chemotherapies on the risk of radiotherapy-induced SMN. In view of the small numbers of cases, SMN types are often pooled for calculating the level of risk. Already in 1976 D’Angio et al. observed that actinomycin-D reduced the risk of radiotherapy-induced SMN in a pooled cohort of patients with various primary cancers. No such effect was seen for cyclophosphamide, vinca alkaloids and antifolic agents [48]. The protective effect of chemotherapy (all forms of therapy combined) on radiotherapy-induced SMN was also observed for cumulative SMN incidence among survivors in excess of 20 years of the childhood cancer survivor study (CCSS) cohort [17]. However, several studies reported the reverse effect. A nested study on the (CCSS) cohort with focus on therapy-induced breast cancer showed that the combination of anthracyclines and radiation potentiates the risk of SMN as compared to radiotherapy alone [16]. A potentiating effect of doxorubicin was observed for radiotherapy-induced SMN among Wilms’ tumour patients [49]. A pooled analysis of patients with various primary malignancies showed that chemotherapy potentiated the risk of radiotherapy-induced SMN overall, with concomitant treatments having a stronger effect than sequential treatments [50]. A similar potentiating effect of chemotherapy was observed for the risk of radiotherapy-induced secondary leukaemia after childhood cancer treatment [51]. Obviously, there is not a single pattern of how chemotherapy modifies the risk of radiotherapy-induced SMN. 

It should be noted that increased death of normal cells from cisplatin plus radiotherapy can led to adverse acute and late normal tissue toxicities. Indeed, it was reported that application of cisplatin in combination with radiotherapy in cervical cancer patients resulted in increased, albeit weak, level of toxicity [52]. Leukopenia results in impaired immune function which may also influence therapy toxicity and its curative outcome [53]. 

How far does chemotherapy alone induce SMN? Here the results are also controversial. In many studies the numbers of cases are low resulting in lack of statistical power. A recent metanalysis revealed that treatment with cisplatin is not associated with a significantly increased risk of SMN [13]. More recent studies on the CCSS cohort revealed that high doses of cisplatin, alkylating agents and anthracyclines can lead to SMN [16,17]. In addition, results from the Dutch Childhood Cancer Oncology Group show that doxorubicin increases the risk of subsequent solid cancers and cyclophosphamide increases the risk of subsequent sarcomas [14]. On the other hand, chemotherapy of adult breast cancer patients leads to a reduction of SMN risk [54,55], as does chemotherapy for Hodgkin disease [56,57]. 

Given the multitude of factors that promote cancer formation [58,59] it is not surprising that the sole activity of chemotherapy and its interaction with radiotherapy can have various effects on normal tissue of patients, depending on the type and dose of therapy, location of the primary cancer and the individual patient response. That chemotherapy can induce SMN can be explained by the mutagenic action of the drugs leading to cell transformation [60] and by its inhibitory effect on the immune system leading to loss of cancer immunosurveillance [61]. That it can reduce the risk of SMN can be explained by the elimination of radiation-damaged cells or—as in the case of breast cancer—by ovarian ablation leading to modified hormonal status of the survivors [57]. 

In view of the above the lack of clear epidemiological evidence for a protective effect of chemotherapy against radiotherapy-induced SMN does not contradict the conclusion of the present study that chemotherapy can reduce the risk of SMN due to an overkill effect. What is the practical implication of this finding? As stated in the introduction, an important aim of research is to quantify the dose–response relationship of SMN. Although linear dose–response relationships for SMN are often derived from epidemiological studies [62] the precise allocation of a dose to the site of a SMN is associated with very large uncertainty due to a dose gradient around the planned treatment volume [9]. Indeed, investigations on the location of SMN with respect to the therapeutic dose received by a primary tumour failed to show a clear dose–response relationship [7,8]. However, the risk of SMN should be included in treatment planning systems and to this end the knowledge of the dose–response relationship is necessary. Of the three parallel models that describe the relationship between the radiotherapy dose to the normal tissue and the risk of cancer: the linear model—assuming a constant increase of risk with dose, the plateau model—assuming a plateau effect, and the competition model—assuming a decline of risk at high doses [5,6], the current results tend to support the latter model, at least for the situation of combined treatment by radiotherapy and chemotherapy. 

Currently there is high interest is identifying biomarkers of individual response to ionising radiation [63,64,65]. We did not correlate the MN frequencies observed in PBL of R and RC patients with any therapy outcome so we cannot make any statement about the suitability of the assay for predicting individual response to radiation. However, the fact that we collected PBL repeatedly and irradiated them ex vivo allowed us to check if patients exhibited a consistently high or low phenotype of individual radiation response at the level of MN in PBL. The results were negative in that a high level of MN at one blood collection point did not significantly correlate to a high level at another time point and vice versa. This result suggests that the variation in radiation-induced cytogenetic damage in PBL does not reflect the intrinsic radiosensitivity of a donor but is more likely influenced by environmental factors. This result fits well with the findings of others [63,66,67]. 

Three of the RC patients received CSF during the course of therapy: RC1 during week 5, RC6 during weeks 2 and 3 and RC8 during week 4. CSF are given when the white blood counts drop below a critical level and they induce a replenishment of differentiated leukocytes by cells from a pool of stem cells. This process of differentiation involves intensive cell division [68] which should lead to a decline of the therapy-induced MN frequency in PBL because chromosomal aberrations and MN are lost during cell division [69]. In an earlier investigation we suggested that the application of CSF reduces the frequency of MN in PBL of patients treated by chemotherapy for lung and ovarian cancer [70]. On the other hand, the intense proliferation and selection against non-damaged cells could reduce the sensitizing effects of chemotherapy on MN induced both by radiotherapy and the ex vivo irradiation. It is interesting to note that from among the 3 patients treated with CSF, patient RC6 who received treatment earliest, showed the steepest increase of MN induced by therapy and the 2 Gy ex vivo dose, accompanied by the steepest decline of proliferation and a less consistent apoptosis response. Patient RC1 who received CSF at the latest time point of therapy (and the highest tumour cure dose) showed the highest frequency of therapy and 2 Gy-induced MN at the 5/6 week blood draw. The impact of CSF may be masked by other factors which modify the response of PBL to therapy and ex vivo radiation, and the low number of patients treated by CSF in this study precludes the possibility of any firm conclusions regarding its influence on radiotherapy-induced MN in PBL. 

## 5. Conclusions

In conclusion the results indicate that cisplatin may positively interact with radiotherapy leading to selective elimination of cells carrying DNA damage, possibly reducing the risk of radiotherapy-induced SMN.

## Figures and Tables

**Table 1:** Age and treatment details of RC and R patients.

Code	Age (Years)	FIGO Stage	Total Dose/Number of Fractions/Technique	Field Number/Mean Field Size (cm^2^)	CDDP Cycles	CSF
RC1	54	III	50.4 Gy/28 fractions + 5.4 Gy/3 fractions/3D-B	3/344 ± 48	4	yes
RC2	53	IIB	50.4 Gy/28 fractions/3D-MLC	5/373 ± 190	5	no
RC3	43	IIB	50.4 Gy/28 fractions/3D-B	5/312 ± 145	4	no
RC4	71	IIB	50.4 Gy/28 fractions/3D-B	4/185 ± 168	4	no
RC5	54	IB	50.4 Gy/28 fractions/3D-B	3/376 ± 41	4	no
RC6	53	IB	50.4 Gy/28 fractions/3D-MLC	5/235 ± 172	4	yes
RC7	76	IIB	50.4 Gy/28 fractions/3D-B	4/267 ± 143	3	no
RC8	52	I	50.4 Gy/28 fractions/3D-B	4/262 ± 146	4	yes
RC9	54	III	50.4 Gy/28 fractions/3D-MLC	4/328 ± 173	5	no
R1	59	II	46.0 Gy/23 fractions/3D-MLC	6/188 ± 130		
R2	66	I	46.0 Gy/23 fractions/3D-B	5/188 ± 130		
R3	63	I	46.0 Gy/23 fractions/3D-B	5/186 ± 144		
R4	63	IB	46.0 Gy/23 fractions/3D-B	4/274 ± 163		
R5	67	I	46.0 Gy/23 fractions/3D-B	4/239 ± 57		
R6	76	I	50.0 Gy/25 fractions/3D-MLC	5/255 ± 204		
R7	55	III	50.0 Gy/25 fractions/3D-MLC	5/255 ± 196		
R8	68	I	46.0 Gy/23 fractions/3D-MLC	6/179 ± 116		
R9	44	I	50.0 Gy/25 fractions/3D-MLC	5/308 ± 168		

RC: cervical cancer patients who received radiotherapy plus chemotherapy; R: endometrial cancer patients who received radiotherapy alone; 3D-B: conformal radiotherapy with block collimators; 3D-MLC: conformal radiotherapy with multileaf collimators; ±: standard deviation; CDDP: cisplatin; CSF: colony stimulating factors.

## Data Availability

The study did not generate any data other than numerical results that are reported in the manuscript.
